# A Rare Case of Amyotrophic Lateral Sclerosis With Asymmetrical Phrenic Nerve Lesion Presenting With Acute Respiratory Failure as an Initial Manifestation

**DOI:** 10.7759/cureus.12547

**Published:** 2021-01-07

**Authors:** Rubén Blanco, Jessie Pichardo, Hassan Abdullah

**Affiliations:** 1 Neurology, Pontificia Universidad Católica Madre y Maestra, Santiago, DOM; 2 Medicine, Pontificia Universidad Católica Madre y Maestra, Santiago, DOM; 3 Neurology, University of Alabama, Birmingham, USA; 4 Medicine, Nishtar Medical University, Multan, PAK

**Keywords:** motor neuron disease, acute hypoxemic respiratory failure, amyotrophic lateral sclerosis, phrenic nerve, upper motor neuron lesion, lower motor neuron lesion

## Abstract

Amyotrophic lateral sclerosis (ALS) is a progressive neurodegenerative disorder that causes muscle weakness, disability, and eventually, death. Respiratory failure is the leading cause of death in ALS. It is common in the advanced stages of the disease. However, acute respiratory failure is a presenting symptom in only a small number of patients, such as in our case. Here, we present the case of a 54-year-old woman with ALS presenting with respiratory failure due to unilateral diaphragm paralysis as the first manifestation. Although rare, respiratory muscle function failure can be the first symptom of motor neuron disease. Therefore, a motor neuron disease such as ALS, which leads to respiratory muscle weakness and diaphragm paralysis, should be considered in cases of unexplained acute respiratory failure.

## Introduction

Amyotrophic lateral sclerosis (ALS), also known as Lou Gehrig’s disease, is a progressive neuromuscular condition characterized by weakness, muscle wasting, fasciculations, and increased reflexes [1]. It is the most common degenerative disease of the motor neuron system. There appears to be a gender predilection for ALS with higher propensity for males [1]. ALS has an annual incidence of 1.75/100,000 person/years, and it is believed to be the same worldwide, most of which is sporadic [2].

Clinical features of ALS are the combination of both upper and lower motor neuron lesion signs and symptoms. Initially, it manifests as problems with dexterity or gait resulting from muscle weakness. Later, patients develop severe, progressive muscular weakness and other symptoms caused by loss of function in both upper and lower motor neurons during the disease. In advanced stages, diaphragm palsy is common and is a primary cause of morbidity [1]. Nevertheless, acute respiratory failure as a presenting symptom is rare.

## Case presentation

**Figure 1:**
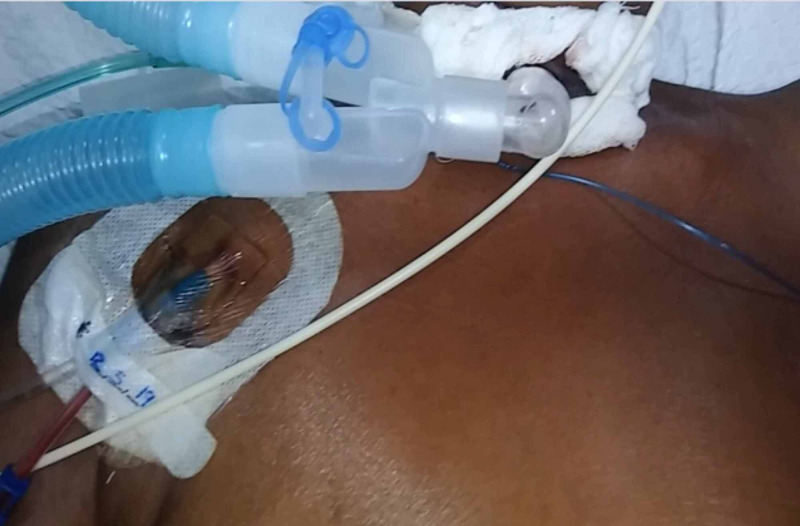
Patient on mechanical ventilation.

We report the case of a 54-year-old woman with ALS presenting with respiratory failure requiring immediate mechanical ventilation (Figure 1).

Apart from having diabetes mellitus, she had a non-significant past medical history. Before this episode, there was no history of body weakness, speech dysfluency, respiratory distress, or sensory deficits. Her neurological examination revealed dysarthria, paraparesis, and distal atrophy. She also had fasciculations, and her plantar reflexes were extensor bilaterally. The sensory system was intact.

**Figure 2:**
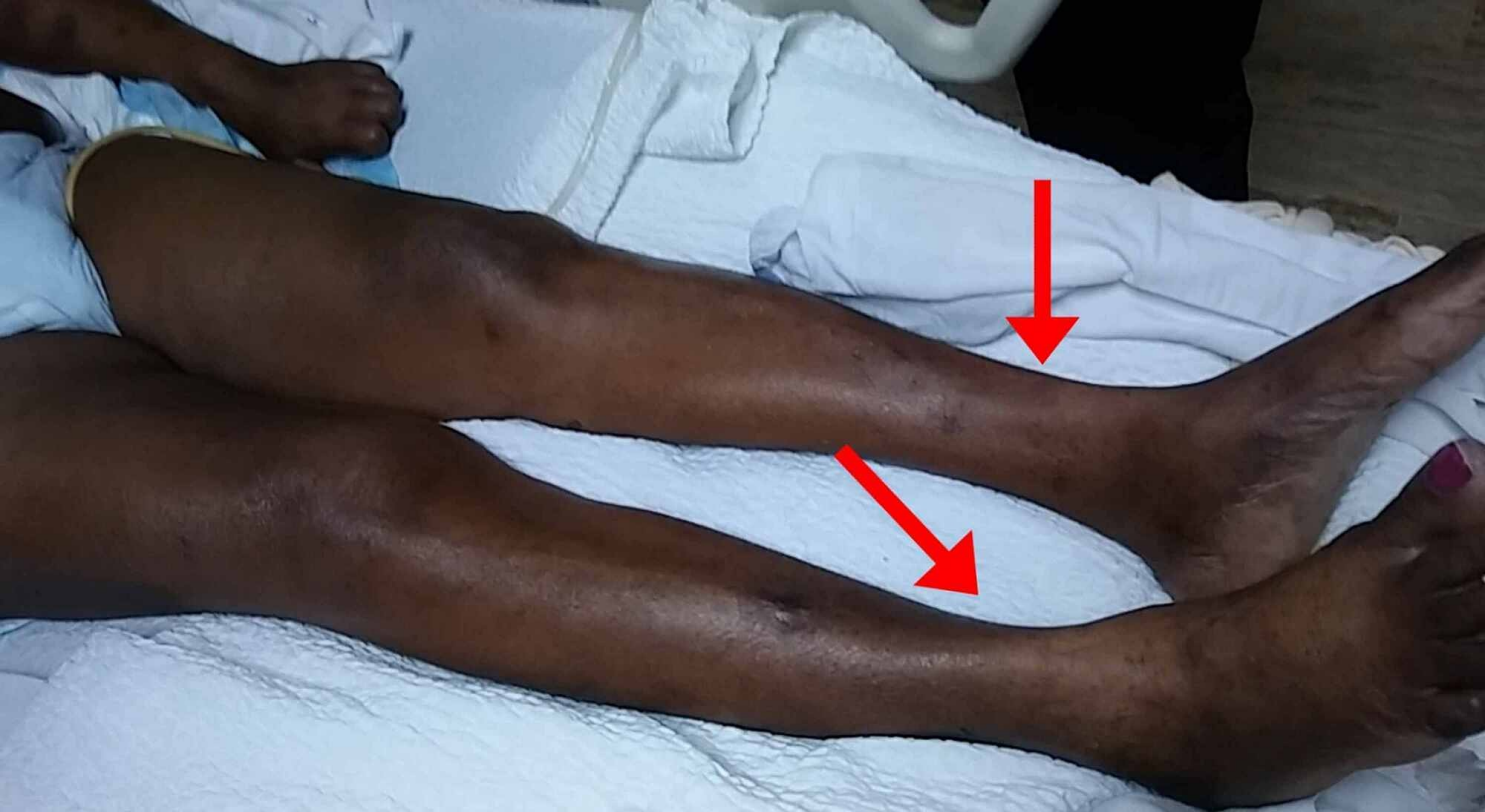
Distal atrophy of the leg muscles bilaterally.

After hospitalization, weaning mechanical ventilation attempts were ineffective as the patient rapidly entered periods of hypoxemia (desaturation). At that time, the doctors on the medicine floor consulted the neurology team. The neurology team initially thought that the patient might have myasthenia gravis (weakness and fatigue of voluntary muscles), but her neurology examination documented distal muscle atrophy (Figure 2).

Hence, we ordered an electroneuromyography for the patient that showed axonal loss without motor conduction blocks, with preserved sensory responses (Table 1).

**Table 1:** Motor nerve conduction studies. ADM, abductor digiti minimi; ADQP, abductor digiti quinti pedis; AH, adductor hallucis; APB, abductor pollicis brevis; EDB, extensor digitorum brevis; L, left; R, right

Nerve	Muscle	Latency	Amplitude	Rel Amp	Duration	Segments	Distance	Lat Diff	Velocity
		Ms	mV	%	ms		Cm	ms	m/s
R median – APB									
Wrist	APB	3.68	2.9	100	5.63	Wrist – APB	7		
Elbow	APB	11.04	2.7	94.7	5.57	Elbow – Wrist	25	5.36	47
L median – APB									
Wrist	APB	3.84	0.2	100	12.71	Wrist – APB	7		
Elbow	APB	10.05	0.2	100	65.73	Elbow – Wrist	25	5.21	48
R ulnar – ADM									
Wrist	ADM	3.13	3.4	100	6.20	Wrist – ADM	7		
B. Elbow	ADM	8.13	3.4	101	6.09	B. Elbow – Wrist	25	5.00	50
A. Elbow	ADM	11.25	3.2	93	5.99	A. Elbow – B. Elbow	16	3.13	51
Axilla	ADM	13.59	2.8	87.1	6.20	Axilla – A. Elbow	11	2.34	47
Erb’s Pt	ADM	15.05	2.6	92.8	6.20	Erb’s Pt – Axilla	35	1.46	240
R ulnar – ADM (1)									
Wrist	ADM	3.13	4.1	100	6.15	Wrist – ADM	7		
Post exercise	ADM	3.65	3.8	92.2	5.47	Post exercise – Wrist		0.52	
R deep peroneal – EDB									
Ankle	EDB	3.44	2.5	100	4.64	Ankle – EDB	8		
Fib Head	EDB	10.57	2.2	81.5	4.58	Fib head – ankle	35	7.14	49
Knee	EDB	11.82	2.2	102	4.79	Knee – fib head	10	1.25	80
R tibial (knee) – AH									
Ankle	AH	5.10	3.9	100	8.13	Ankle – AH	8		
Knee	AH	14.74	3.6	90.7	7.66	Knee – Ankle	42	9.64	44
L tibial (knee) – AH									
Ankle	AH	5.94	3.7	100	7.34	Ankle – AH	8		
Knee	AH	17.60	3.5	95.2	10.83	Knee – Ankle	42	11.67	36
L deep peroneal – EDB									
Ankle	EDB	5.10	0.5	100	3.91	Popliteal fossa – Gastronec	8		
Fib H	EDB	13.59	0.4	75.2	8.65	Knee – Popliteal fossa	35	8.49	41
Knee	EDB	15.52	0.5	113	5.52	Ankle – ADQP			

**Figure 3:**
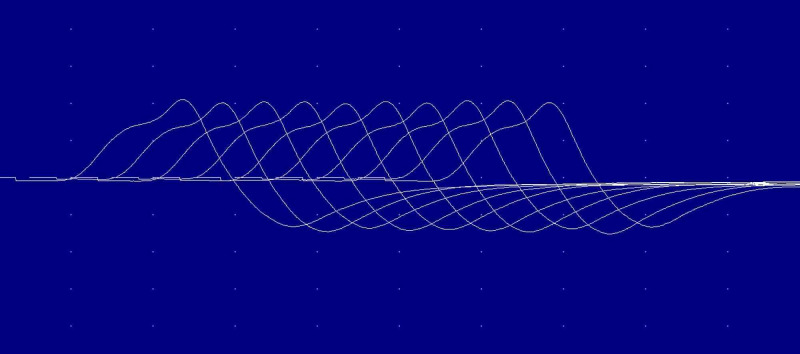
Repetitive nerve stimulation on right ADM. ADM, abductor digiti minimi

The repetitive nerve stimulation was normal at abductor digiti minimi bilaterally that ruled out any neuromuscular junction dysfunction such as myasthenia gravis (Figure 3).

**Figure 4:**
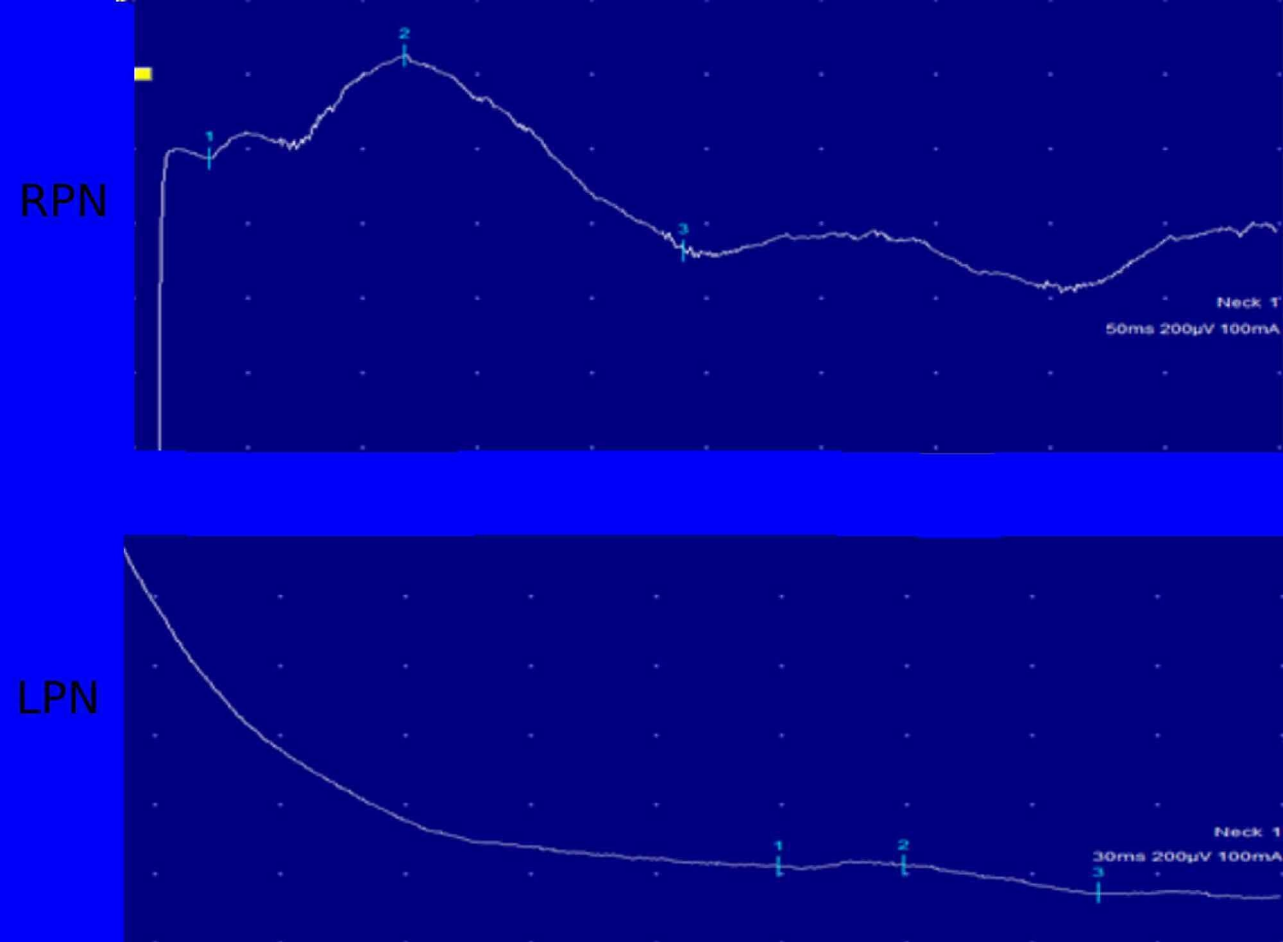
Electroneuromyography of the right and left phrenic nerves. LPN, left phrenic nerve; RPN, right phrenic nerve

The conduction velocity performed in the phrenic nerves bilaterally showed an abnormal left-sided response (Figure 4).

The left phrenic nerve response had increased latency and decreased amplitude (Table 2).

**Table 2:** Amplitude and latency of the right and left phrenic nerves.

Stimulus site	Lat (ms)	Amp (mV)
Left phrenic nerve	17.92	0.1
Right phrenic nerve	7.25	0.5

**Figure 5:**
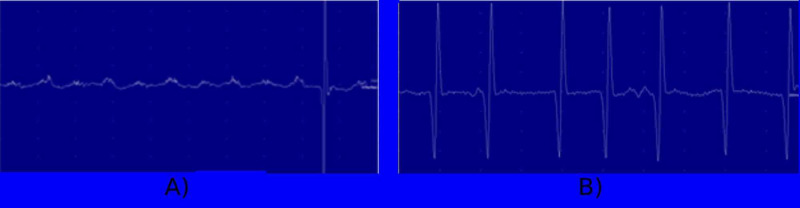
Fasciculations as a sign of denervation (A), and discrete recruitment pattern in deltoid muscle (B).

The needle examination revealed signs of denervation, as shown in Figure 5A, in all the muscle territories evaluated, including the bulbar area, signs of chronic reinnervation, and reduced discrete motor unit recruitment pattern (Figure 5B).

These results were compatible with a motor neuron degenerative syndrome, that is, ALS, along with a concomitant left phrenic nerve lesion. Hence, the patient was diagnosed with ALS accompanied with a partial peripheral lesion of the phrenic nerve.

**Figure 6:**
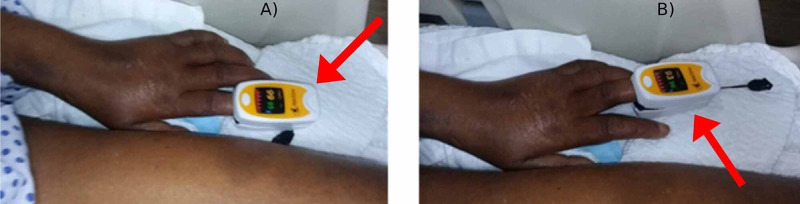
(A) Patient on mechanical ventilation (99% saturation). (B) One minute after weaning from the ventilator (93% saturation).

We did the test of weaning her off from mechanical ventilation, and 30 seconds to a minute later, her saturation reduced to 93% (Figure 6).

This confirmed the phrenic nerve lesion. Any mechanical cause of the phrenic nerve lesion was ruled out on a chest X-ray and chest computed tomography. We concluded that she was desaturating due to paralysis of the left hemidiaphragm and the rib cage muscle weakness secondary to the underlying disorder.

## Discussion

ALS is caused by the progressive loss of motor neurons, leading to weakness and subsequent paralysis of the face, bulbar, and extremity muscles, including the diaphragm [3].

Respiratory failure has been mostly described in the late stages of the disease; however, our patient revealed an atypical presentation in isolation in the acute phase, as described in other studies [4]. Czapliński et al. reported a case presenting respiratory failure as an early manifestation of ALS [5]. Similar to our case, the patient had diaphragmatic paralysis, but it was bilateral instead of unilateral. Crescimanno et al. also noted early and rapidly progressing respiratory failure in a patient with ALS [6].

Patients who develop acute respiratory failure may have an underlying comorbid condition, for example, chronic obstructive pulmonary disease (COPD). Fromm et al. [7] and Parhad et al. [8] observed acute respiratory failure as a sole initial manifestation in patients later diagnosed with ALS. But these patients had COPD as a comorbidity. Similarly, Al-Shaikh et al. reported a case with similar signs and symptoms, with pneumothorax as a background illness [9].

When respiratory disturbance is the sole manifestation, more significant denervation signs have been described in the diaphragmatic muscles than in the rest of the skeletal muscles [4]. The most common cause of diaphragmatic paralysis in adults is trauma to the cervical spine, followed by neuromuscular diseases such as myasthenia gravis, myopathies, dystrophies, and medullary anterior horn disease, as in the present case [10].

## Conclusions

ALS is a complex disease in which patients are highly dependent on medical care. Acute respiratory failure can be the first symptom of motor neuron disease. Therefore, in cases of unexplained acute respiratory failure or when there is prolonged intubation for no apparent reason, a motor neuron disease such as ALS should be considered. ALS can lead to respiratory muscle weakness and diaphragm paralysis. Respiratory evaluation and continuous monitoring can prolong survival and improve the quality of respiratory care for patients. The initial electrophysiological evaluation continues to be the best access and accurate diagnostic tool when a neuromuscular disorder is suspected as the genesis of acute dyspnea.
